# Smoking Status and Metabolic Syndrome in the Multi-Ethnic Study of Atherosclerosis. A cross-sectional study

**DOI:** 10.1186/1617-9625-10-9

**Published:** 2012-06-20

**Authors:** Ivan Berlin, Susan Lin, Joao A C Lima, Alain Gerald Bertoni

**Affiliations:** 1 Hôpital Pitié-Salpêtrière, Assistance publique-Hôpitaux de Paris, Université P. & M. Curie, Faculté de médecine, INSERM 894, Paris, France; 2 Center for Family and Community Medicine, Columbia University, New York, NY, USA; 3 Johns Hopkins University School of Medicine, Baltimore, MD, USA; 4 Wake Forest University School of Medicine, Winston-Salem, NC, USA

**Keywords:** Metabolic syndrome, Smoking, Ethnic groups, Body mass index

## Abstract

**Background:**

Current smoking is associated with type 2 diabetes mellitus and impaired glucose tolerance but its association with the metabolic syndrome (metS), particularly with sufficiently sampled African American representation, has not been clearly established.

**Objective:**

To assess whether a) metS is associated with smoking; b) any increased risk of metS among smokers is independent of body mass index (BMI) compared with non-smokers; c) smoking status is differentially associated with the metS and its components across different ethnic groups.

**Methods:**

Cross sectional analysis of the Multi-Ethnic Study of Atherosclerosis (MESA) a community population-based sample free of cardiovascular disease.

**Results:**

Current smokers (N = 769) had higher risk of metS (odds ratio [OR, 95% confidence interval]: 1.4, 1.1-1.7) versus never (reference, N = 2981) and former smokers (1.0, 0.8-1.1, N = 2163) and for metS components: high waist circumference (WC) (OR:1.9, 1.2-2.1), low high density lipoprotein cholesterol (HDL-C) (1.5, 1.3-1.8), elevated plasma triglycerides (TG) (OR:1.4, 1.2-1.7) as well as high C-reactive protein (CRP, an inflammatory marker) concentration (OR: 1.6,1.3-2.0) compared to never and former smokers after adjustment for BMI. A smoking status by ethnicity interaction occurred such that African American current and former smokers had greater likelihood of low HDL-C than White counterparts.

**Conclusions:**

This study found that smoking is associated with the metS and despite the lower BMI of current smokers the prevalence of low HDL-C, elevated TG and CRP is higher among them than among non-smokers. African Americans generally have higher HDL-C than Whites but smoking wipes out this advantage.

Multi-Ethnic Study of Atherosclerosis (MESA) ClinicalTrials.gov Identifier: NCT00005487

## Introduction

Tobacco use continues to be the leading global cause of preventable death. It kills nearly 6 million people and causes hundreds of billions of dollars of economic damage worldwide each year [1]. Cigarette smoking causes about 1 of every 5 deaths in the United States each year [2]. The leading causes of death from smoking are cardiovascular diseases (1.69 million deaths), chronic obstructive pulmonary disease (0.97 million deaths) and lung cancer (0.85 million deaths) [3]. Smoking cessation leads to reduced mortality, in particular, in patients with coronary heart disease [4]. Active smoking increases the prevalence and incidence of type 2 diabetes mellitus [5-8] and glucose intolerance [9] as does secondhand smoke exposure [9,10]**.**

Smoking is associated with increased likelihood of low HDL-C [11-13]; and has been suggested to be associated with insulin resistance [14-16] and increased level of inflammatory markers (e.g., CRP) [17,18]. Some studies have assessed the smoking – metS relationship [19-24] but we are not aware of data on the association of smoking status with the clustered metabolic risk factors known as metabolic syndrome and its components such as increased TG, reduced HDL-C concentrations, increased blood pressure (BP) and impaired fasting glucose (IFG) in multi-ethnic groups.

It is well known that cigarette smokers weigh less than non-smokers [25-30] and their age and gender adjusted body mass index (BMI) is on average 1 kg/m^2^ less than that that of non-smokers [31]. Despite this, compared to non-smokers, current smokers are more likely to have abdominal type obesity [24,26,29]. The lower BMI of smokers compared to non-smokers raises questions regarding the impact of smoking on cardiovascular disease (CVD) risk factors such as the metS, its components and inflammatory markers such as CRP [32].

Therefore the aims of this cross sectional analysis were a) to assess metabolic syndrome, its components and a CVD risk factor CRP among smokers compared to former and never smokers; b) to test the contribution of BMI on the associations between smoking status and these risk factors; and c) to evaluate whether the association of smoking status with these risk factors varies across ethnicity and secondarily by gender.

## Methods

### Study population

The Multi-Ethnic Study of Atherosclerosis (MESA) is a multicenter cohort study of participants recruited between 2000 and 2002; details regarding recruitment and design have previously been published [33]. Briefly, it is a population-based sample of men and women aged 45–84 who identified themselves as non-Hispanic White, African-American, Hispanic or Chinese American and were free of clinically apparent cardiovascular disease. Participants were recruited from six US communities: Baltimore City and County, MD, Chicago, Ill, Forsyth County, NC, Los Angeles County, CA, northern Manhattan and the Bronx, NY, and St Paul, MN. The institutional review boards of all participating centers approved the study and all participants gave informed consent. From the total sample (N = 6814) we excluded participants with missing smoking status or missing other data precluding the characterization of metabolic syndrome. We excluded participants with diabetes, or taking medicines for diabetes or having fasting glucose >125 mg/dL, and those whose diabetes status was unknown. Thus, the study population consisted of 5913 participants: 37.6% non-Hispanic Whites, 23.6% African Americans, 10.7% Chinese Americans and 28.1% Hispanics.

### Measures

At baseline, questionnaires were used to obtain information about demographics, socioeconomic status, medical history and medications. Waist circumference at the umbilicus was measured to the nearest 0.1 cm using a steel measuring tape. Height and weight was measured by a stadiometer and calibrated scale. Body mass index was calculated from height and weight as kg/m^2^. Resting blood pressure was measured three times with participants in a seated position with a Dinamap model Pro100 automated oscillometric sphygmomanometer (Critikon); the average of the last two measurements was used in the analysis. Triglycerides, HDL-C, blood glucose, and plasma insulin concentrations were measured from blood samples obtained after a 12-hour fast. Metabolic syndrome was classified using the updated Adult Treatment Panel III (ATP III) definition [34] as three or more of the following: High WC (WC > 102 cm for men and WC > 88 cm for women); elevated TG (≥150 mg/dL); low HDL-C (men < 40 and women < 50 mg/dL); elevated blood pressure (systolic blood pressure ≥130 or diastolic blood pressure ≥85 mmHg or use of medications for hypertension); and elevated fasting glucose (≥100 mg/dL). For Chinese Americans we used the International Diabetes Federation (IDF) cut off point for high WC: >90 cm for men and >80 cm for women [35]. We defined impaired fasting glucose as between 100 mg/dL and125 mg/dL. Insulin resistance was estimated by homeostasis model assessment of insulin resistance (HOMA-IR), calculated according to the formula (insulin (mU/I)*(glucose [mg/dL]0.055)/22.5). The top quartile value was used as cutoff point to determine insulin resistance. CRP was measured using the BNII nephelometer (N High-Sensitivity CRP); intra-assay coefficient of variation for CRP range from 2.3 to 4.4% and inter-assay coefficients of variation range from 2.1 to 5.7% [36]. Elevated CRP was defined as CRP ≥ 5 mg/L [37]. Physical activity was measured by using a detailed, semiquantitative questionnaire adapted from the Cross-Cultural Activity Participation Study [38]. The sum of minutes spent in all activity types was multiplied by the metabolic equivalent (MET) level and physical activity level was expressed as min/week*MET [39].

Never smoking was defined as lifetime consumption of less than 100 cigarettes (N = 2981). There were 2163 former smokers. Among them, 2105 quit smoking ≥ 1year earlier and 58 between 30 days and 1 year. These latter were added to the group of former smokers. The mean pack years was 21 (SD = 25) for former and 27 (SD = 32) for current smokers. Data were analyzed according to smoking status and not according to pack years for two reasons. First, use of pack years does not provide information about former smoking status and can erase the effect of quitting. Second, a body of tobacco research has suggested that smoking induced disease risk, in particular cardiovascular risk, is dependent to a greater degree on the duration of exposure, and less dependent on the amount smoked (i.e. low number of cigarettes per day or occasional cigarette smoking can also be associated with increased disease risk) [40].

Alcohol use was screened with the following questions: “Have you ever consumed alcoholic beverages?” and “Do you presently drink alcoholic beverages?” and participants were classified as current, former or never users.

### Data analysis

ANOVA and chi-square tests were used to examine the characteristics of the study participants by smoking status (never, former and current smoker). Logistic regression models were used to examine the association of smoking status with CVD risk factors with and without adjustment for BMI. The following factors were included as covariates: age, gender, ethnicity, site, household income, alcohol consumption, physical activity, lipid lowering and antihypertensive drugs and education. Interactions of race/ethnicity and gender with smoking status were tested for each CVD risk factor. Data are reported as frequencies and odds ratios (OR) with 95% confidence intervals (CI) if otherwise not indicated. Significance level was set at p ≤ 0.05.

## Results

Current smokers were younger, and reported less household income and education than never smokers. Current smoking was the most frequent among non-Hispanic Whites and the less frequent among Chinese Americans. Use of antihypertensive medications or lipid lowering drugs were less frequent among current smokers than among never or former smokers and they reported more moderate/vigorous physical activity.

The prevalence of ATP-III metS was similar between the three smoking status groups. However, when looking at the individual metS components, the prevalence of all risk factors except IFG and HOMA-IR were significantly different between smokers, former smokers and never smokers. In particular, while among current smokers high WC, above normal BMI (overweight/obesity), and elevated BP were less frequent, current smokers had higher prevalence of low HDL-C, high plasma TG and elevated CRP (Table 1).

**Table 1:** Characteristics of the study population by smoking status

	**Never smoker**	**Former smoker**	**Current smoker**	**P-value**
	**N = 2981**	**N = 2163**	**N = 769**	
**Demographics**				
Age (years)	61.8 (10.6)	63.2 (9.9)	57.8 (9.2)	<0.001
Gender-Males (%)	37.7	56.9	50.8	<0.002
Race/ethnicity (%)				<0.001
Non-Hispanic White	36.4	50.0	36.8	
Chinese American	17.7	6.1	4.7	
African American	23.3	26.1	36.3	
Hispanic	22.5	17.9	22.2	
Education (%)				<0.001
High school or less	36.5	29.9	38	
Some college	24.8	30.8	37.8	
Bachelor or graduate	38.7	39.3	24.2	
Household income level per year (%)				<0.001
<$25000	31.9	26.0	31.5	
>$25000 and < $50000	28.2	27.7	32.8	
>$50000 and < $75000	16.4	18.6	18.4	
>$75000	23.4	27.7	17.3	
**Medications**				
Use of antihypertensive medications (%)	30.7	30.4	23.4	<0.001
Use of lipid lowering medications (%)	14.1	16.3	10.7	<0.001
**Behavioral variables**				
Alcohol use (%)				<0.001
Never	32.4	6.2	8.2	
Former	18.2	28.4	22.5	
Current	49.4	65.4	69.3	
Physical activity (min/week-MET)	5604 (5625)	5857 (6095)	6631 (6662)	<0.001
First quartile <2055	26.0	23.2	25.6	<0.001
2nd quartle (2055–4125)	25.4	26.8	19.0	
3rd quartile (4125–7545)	24.8	25.7	23.9	
4th quartile (>7545)	23.8	24.3	31.5	
**Cardiometabolic profile** (%)				
ATP-III Metabolic syndrome	26.5	26.3	27.3	0.9
*Metabolic syndrome components*				
High WC	58.2	53.5	51.6	<0.001
Elevated BP	50.9	54.8	44.0	<0.001
Low HDL-C	34.1	31.4	42.0	<0.001
Elevated TG	27.6	26.1	32.3	0.005
Impaired fasting glucose	15.7	16.3	14.7	0.6
Insulinresistance (HOMA-IR)	25.5	26.6	23.6	0.3
Elevated CRP	18.1	19.5	24.0	0.001
Normal weight (BMI < 25)	32.7	27.3	30.9	<0.001
Obese (BMI > 29.9)	29.0	31.6	28.2	0.08

Data are means (SD) or percentages.

Table 2 shows the associations of smoking status with metS and its components with and without adjustment for BMI. Smoking status was significantly associated with the presence of metS or high WC only in the models adjusted for BMI. Without adjustment for BMI, HOMA-IR was significantly less frequent among current smokers than among former or never smokers but the differences disappeared after adjustment for BMI (Table 2).

**Table 2:** Effect of body mass index (BMI) on the association of cardiovascular disease risk factors with smoking status

	**BMI unadjusted OR**			**p-value**	**BMI adjusted OR**			**p-value**
	**Never smokers**	**Former smokers**	**Current smokers**		**Never smokers**	**Former smokers**	**Current smokers**	
Metabolic syndrome	reference	1.0 (0.9-1.2)	1.1 (.9-1.3)	0.5	reference	1.0 (0.8-1.1)	**1.4 (1.1-1.7)**	**0.03**
*Metabolic syndrome components*								
High WC	reference	1.1 (0.9-1.2)	0.9 (0.7-1.1)	0.2	reference	1.1 (0.9-1.3)	**1.6 (1.2-2.1)**	**0.004**
Elevated BP	reference	1.1 (0.9-1.2)	0.9 (0.7-1.0)	0.09	reference	1.0 (0.9-1.2)	0.9 (0.8-1.1)	0.5
Low HDL-C	reference	1.0 (0.9-1.1)	**1.3 (1.2-1.6)**	**0.001**	reference	1.0 (0.8-1.1)	**1.5 (1.3-1.8)**	**<0.001**
Elevated TG	reference	1.0 (0.9-1.1)	**1.1 (1.1-1.6)**	**0.009**	reference	1.0 (0.8-1.1)	**1.4 (1.2-1.7)**	**<0.001**
Impaired fasting glucose	reference	1.0 (0.8-1.1)	1 (0.8-1.2)	0.9	reference	0.9 (0.8-1.1)	1.1 (0.9-1.4)	0.2
Insulin resistance (HOMA-IR)	reference	1.1 (0.9-1.2)	**0.8 (0.7-0.98)**	**0.02**	reference	1 (0.9-1.2)	1.0 (0.8-1.2)	0.9
Elevated CRP	reference	**1.2 (1.03-1.4)**	**1.3 (1.1-1.6)**	**0.01**	reference	1.2 (0.98-1.4)	**1.6 (1.3-2.0)**	**<0.001**

Adjusted for age, gender, ethnicity, site, household income, alcohol consumption, physical activity, lipid lowering and antihypertensive drug and education.

OR are shown with one decimal for clarity.

Data are odds ratios (OR) and (95% confidence intervals).

The significant association of low HDL-C, elevated TG and elevated CRP with smoking status did not change after adjusting for BMI (Table 2).

Table 3 shows the significant interactions of smoking status with ethnicity and gender. There was a significant interaction of smoking status with ethnic groups for HDL-C. Among African Americans both former and current smokers were more likely than never smokers to have low HDL-C while only current smokers were more likely to have low HDL-C among non-Hispanic Whites or Hispanics. Thus, the significant smoking status by ethnicity interaction results from the higher likelihood to have a higher prevalence of low-HDL-C among former smoker African Americans than among non-Hispanic Whites or Hispanics.

**Table 3:** Differential association of metabolic syndrome components with smoking status by gender and race

	**BMI unadjusted OR**			**p-value**	**BMI adjusted OR**			**p-value**
	**Never smokers**	**Former smokers**	**Current smokers**		**Never smokers**	**Former smokers**	**Current smokers**	
Elevated TG*								
Males	reference	1.0 (0.8-1.3)	**1.4 (1.1-1.8)**	0.055	reference	1.0 (0.8-1.2)	**1.5 (1.1-2.0)**	**0.006**
Females	reference	1.0 (0.8-1.2)	1.3 (0.9-1.7)	0.3	reference	1.0 (0.8-1.2)	1.4 (0.99-1.8)	0.06
Interaction of gender with smoking status				**0.02**				**0.04**
High WC*								
Males	reference	**1.2 (1.0-1.5)**	1.1 (0.9-1.4)	0.1	reference	**1.3 (1.0-1.7)**	**2.2 (1.5-3.3)**	**<0.001**
Females	reference	1.0 (0.9-1.3)	0.8 (0.6-1.0)	0.08	reference	1.0 (0.8-1.3)	1.2 (0.8-1.8)	0.7
Interaction of gender with smoking status				**<0.001**				**<0.001**
Low HDL-C**								
Non-Hispanic Whites	reference	0.8 (0.7-1.0)	**1.3 (1.0-1.7)**	**0.02**	reference	0.9 (0.7-1.0)	**1.5 (1.1-2.0)**	**0.001**
African Americans	reference	**1.5 (1.1-1.9)**	**1.6 (1.1-2.2)**	**0.007**	reference	**1.4 (1.1-1.9)**	**1.8 (1.3-2.6)**	**0.001**
Hispanics	reference	1.1 (0.8-1.5)	**1.5 (1.0-2.2)**	**0.01**	reference	1.1 (0.8-1.4)	**1.6 (1.1-2.3)**	0.07
Chinese Americans	reference	0.7 (0.4-1.2)	1.2 (0.6-2.5)	0.3	reference	0.7 (0.4-1.1)	1.1 (0.5-2.3)	0.3
Interaction of ethnicity with smoking status			0.056					**0.026**

*Adjusted for age, ethnicity, site, household income, alcohol consumption, physical activity, lipid lowering and antihypertensive drug and education.

**Adjusted for age, gender, site, household income, alcohol consumption, physical activity, lipid lowering and antihypertensive drug and education.

OR are shown with one decimal for clarity.

Data are odds ratios (OR) and (95% confidence intervals).

There was no statistically significant difference in elevated TG or high WC by smoking status neither among males nor among females. However, when adjustment was made for BMI, male current and former smokers were more likely to have high WC than never smokers. These associations were not statistically significant among females.

## Discussion

This study found that current smoking is associated with the metS and despite the lower BMI of current smokers the prevalence of high WC, low HDL-C, elevated TG is higher than among non-smokers. The prevalence of high CRP, an inflammatory marker associated with CVD risk [32] was also higher among current smokers compared to never or former smokers. The only difference which occurred between smokers of different ethnic origin concerned low HDL-C: the highest risk for low HDL-C was observed among African American current and former smokers.

At any age, both male and female smokers have lower BMI than non-smokers or former smokers [25,27,28,30]. Thus the question arises whether BMI influences or not the presence of metS and its components among persons of different smoking status: never, former or current smoking. When adjusted for BMI, we found that smokers were at higher risk of metS and high WC. Although smokers of this sample seemed to have less frequently insulin resistance than non-smokers (never or former smokers), this apparent benefit was simply related to the confounding effect of their lower BMI. Although adjustment for BMI had no influence on the prevalence of elevated blood pressure and impaired fasting glucose concentration, it revealed that the metS is more frequent among current than among former or never smokers, and that the high WC is associated with current smoking. Smoking seems to be associated with low HDL-C, high TG and CRP independently of BMI.

Conflicting data exist about the smoking status – metS relationship. Data from the Third National Health and Nutrition Examination Survey (NHANES III) showed that when adjusted for all modifiable lifestyle factors current smoking, when compared to never smoking, was associated with increased risk of metS when adjusted for BMI for both men and women [19]. In a Korean population, more than 20 pack-year smoking has been found to be associated with a 1.9 fold risk of BMI adjusted metS [20]. A recent cross sectional study did not find an association between smoking status and metS [21] but no adjustment was made for BMI. Geslain-Biquez et al. [22] reported that the frequency of metS was higher among smokers (22.5%) than among non-smokers (15.3%). In this sample there was no difference in BMI between smokers and non-smokers showing that at equal BMI smoking is associated with increased prevalence of metS. To our knowledge only two prospective studies reported about incident metS and smoking status. Carnethon et al. [23] found when analyzing data of the CARDIA study that baseline smoking status did not predict incident metS during an average follow up of 13.6 years but there is no information what was the percent of smokers who became former smokers which potentially could reduce the risk of developing metS. Although this study demonstrated ethnic differences in developing metS, it did not report on ethnicity by smoking status interaction [23]. In a Turkish population during a mean follow up of 5.9 years smoking was inversely associated with WC and among women smoking reduced the risk of metS by half; however this “protective” effect of smoking on metS was not accompanied by a subsequent reduction in coronary heart disease or all-cause mortality [24].

In the current sample the multivariate analysis showed that smoking status was not associated with blood pressure. The relationship between smoking and blood pressure is controversial some studies reporting higher others lower blood pressure [41]. However, compared to non-smokers, smoking has been more consistently found to be associated with lower systolic and diastolic blood pressure in Nord-American [17], European [24,42] and Asian populations [43,44]. The lower blood pressure found among smokers may be associated with their lower BMI as shown in the present study.

In the present study we did not find in the multivariate analysis that HOMA IR, a proxy measure of insulin resistance, or fasting plasma glucose concentration were associated with smoking status. It has been suggested that the insulin resistance syndrome is the key player between cigarette smoking and CVD [15]. This hypothesis is mainly based on acute human lab studies [14,16] and on the considerations that smoking may result in reduced skeletal muscular blood flow, vascular changes and abdominal type adiposity, all potentially associated with reduced insulin-mediated glucose uptake and insulin sensitivity. However, a large cross sectional study did not confirm that active smoking was associated with increased insulin resistance [45]. Further studies are needed to explore specifically the smoking - insulin resistance relationship.

Because of the demonstrated ethnic and gender differences in the prevalence of the metS and its components [46-50], this study tested whether the association of the metS and its components with smoking varied by ethnicity and secondarily by gender. There was no apparent evidence that the studied CVD risk factors associated with smoking varied significantly by ethnic groups except the finding that low HDL-C was more prevalent among African American current and former smokers compared with non-smokers and their counterparts in other ethnic groups. African Americans generally have higher HDL-C than non-Hispanic Whites. According to the present data smoking is associated with reduction in (the protective) HDL-C in African Americans and may increase by this their CVD risk more than among non-Hispanic Whites. This finding strongly suggests that intensive specific interventions should be targeted among African American smokers and former smokers to improve their smoking induced negative lipid profile to reduce the burden of CVD risk.

The prevalence of metS components and CRP did not differ between males and females according to smoking status suggesting a similar risk factor level among men and women smokers. The unfavourable association of current and former smoking with high WC after adjustment for BMI became significant only among males. It is not clear whether this differential association with gender concerning only WC was related to the intensity of exposure to tobacco or to other factors. Future studies are needed to examine the plausible causes.

There are several limitations to this study. This was a cross sectional analysis; smoking status was based on self-report and no biological validation approaches such as expired air carbon monoxide or saliva/plasma/urine cotinine concentrations were used to confirm self-report of smoking status. BMI is inversely related with the number of cigarettes smoked per day [26]. It has also been shown that HDL-C levels decrease on a dose-dependent way with increases in the number of cigarettes smoked per day [11]. It would have been important to analyze if, among current smokers, these dose–response relationships exist for other metS components and whether they are similar or not across the different ethnic groups or gender. Unfortunately, this could not have been done because of the low power for these analyses: among current smokers cigarettes per day categories by ethnic groups or by gender yielded very few cell numbers. A major limitation is that the cross sectional nature of the data did not allow assessing prospectively changes in metS and its components in particular among smokers who quit.

Because smokers cite control of body weight as a reason to continue smoking [51-55] and the tobacco industry reinforces this notion through advertising [56] it is important to look at the effect of the lower body weight associated with current smoking on CVD risk factors such as metS components and inflammatory markers. This study found that lower BMI of current smokers does not improve their lipid profile and the inflammatory marker CRP. This can partially explain that lower BMI of smokers does not protect against developing smoking related cardiovascular disorders.

In conclusion, smoking is associated with the metS and despite the lower BMI of smokers compared to non-smokers the risk profile of components of metS with demonstrated association with CVD risk is maintained. Among African Americans who generally have higher HDL-C than Whites, smoking nullifies this advantage. Current smoking may differentially be associated with metS components in specific ethnic groups or among males potentially leading to a differentially increased CVD risk.

## Competing interests

Author Disclosure Statement: None of the authors reports competing financial interest in connection with this manuscript. I. Berlin has a tenureship salary from Université P.& M. Curie and Assistance publique-Hôpitaux de Paris. He had no any other funding source supporting this work. He reports having received honoraria for consultancies over the past 5 years from Sanofi-Aventis and Pfizer Ltd. without any relationship with this manuscript. Susan Lin, Joao Lima and Alain G. Bertoni report no conflict of interest. None of the authors declare any non-financial competing interests. All authors declare not having any kind of relationship with the tobacco or alcohol industry.

## Authors’ contributions

IB and SL conceived and drafted the first version of the manuscript. IB, SL and AB drafted the statistical analytical plan. IB and SL analyzed the data. JL participated in the design of the study and helped to draft the manuscript. All authors read and approved the final manuscript.
